# Review on the prevalence, risk factors and disease Management of Hypertension among floating population in China during 1990–2016

**DOI:** 10.1186/s41256-018-0076-9

**Published:** 2018-07-23

**Authors:** Lina Su, Long Sun, Lingzhong Xu

**Affiliations:** School of Public Health, Department of Social Medicine and Health Management, Shandong University, No.44, Culture west road, Lixia District, Jinan, 250012 Shandong Province China

**Keywords:** Hypertension, Floating population, Risk factor, Disease management

## Abstract

**Objective:**

To have a basic and comprehensive understanding about the prevalence, risk factors and disease management situation of hypertension among floating population in China.

**Method:**

We used “(hypertension or hypertensive or chronic disease) and (floating population or migrant worker)” as the key words, to search in the China academic literature database (CNKI), Wan Fang database, PubMed and Web of Science for relevant literature and extracted the data about the prevalence of hypertension, relevant risk factors and disease management of floating population in China from 1990 to 2016.

**Result:**

The 23 related studies that entered into final analysis were all articles in Chinese. The prevalence of hypertension in floating population is lower than that in both general population and local residents. The prevalence of hypertension in male floating population is higher than that in females. In addition, the prevalence of hypertension also increases with age growing. As for the risk factors of hypertension, the rate of drinking in floating population is higher than that in general population and local residents, while the rates of overweight and obesity in floating population are lower than that in general population in China. Finally, the rates of awareness, treatment and control of hypertension are also lower in floating population.

**Conclusion:**

The major problem of floating population is focused on their unhealthy lifestyle (drinking) and deficient disease management. Therefore, we should increase the fund and facility support for public health service system so as to improve their service delivery ability, and enforce the education and unhealthy lifestyle intervention to improve their health awareness and compliance to disease management.

## Background

Floating population is a special group coming with the rapid development of economy and urbanization in China, mainly refers to the adults at the childbearing age who leave their domicile for the purpose of making a living [1]. According to the new statistics, the number of floating population in China has already reached 247 million [2]. Because of low-level education, heavy work and poor living condition, the health status of floating population is always suboptimal, and the prevalence of chronic disease is at a high level [3]. Meanwhile, there also exists vast disparities of health insurance coverage and health service utilization between floating population and local residents, most of the floating population have no timely access to primary or some other kinds of health care service which cause the poor control of chronic diseases.

Hypertension, as one of the major chronic disease, is not only the direct cause of health loss, but also a major and independent risk factor of other cardiovascular diseases including coronary heart disease, heart failure and stroke. Due to the incomplete monitoring system,it is quite difficult for the management of those hypertensive patients in floating population because of the lack of relevant data about prevalence, risk factors and disease management of hypertension in floating population. Therefore, this review was designed to focus on the prevalence, risk factor and disease management of hypertension among floating population in China, so as to provide some references for improvement measures.

## Method

### Data sources

A systematic search was performed using China academic literature (CNKI), Wan Fang, PubMed and Web of Science databases across the period 1990–2016 to identify relevant researches. Search terms used either singularly or in combination were “hypertension”, “hypertensive”, “chronic disease”, “floating population” and “migrant worker” in the thesaurus and index lists of the relevant databases in both Chinese and English words. Also “free text” words were used to supplement the search terms [medical subject heading (MeSH) search terms in the case of Medline]. Manual searches of the bibliographies of searched articles and reviews in the field were also conducted.

### Study selection

The inclusion criteria: (1) randomized clinical trials, prospective and retrospective observational studies; (2) published as original articles in scientific journals; (3) research objects are floating population. The exclusion criteria: (1) review article, questionnaire reliability and validity research; (2) non-Chinese Mainland floating population; (3) the lack of related data. The information table was designed by the research team, and the information was extracted by 2 researchers. The opinions were decided by the task group when the opinions were extracted.

### Quality check

Literature evaluation criteria recommended by the Agency for Healthcare Research and Quality (AHRQ) in the United States were used to evaluate the cross-sectional study quality [4]. The scale consisted of 11 items, including subjects, selection, research, quality control and data processing, using the “yes”, “no” and “unclear” as answers. The quality evaluation was conducted independently by 2 researchers (Lina Su, graduate student, majored in health management. Long Sun, doctor, majored in suicidology), and the decision was made by the senior researcher (Lingzhong Xu, professor, majored in health economy) when differences occurred.

## Results

### Incorporation of literature

**Fig. 1:**
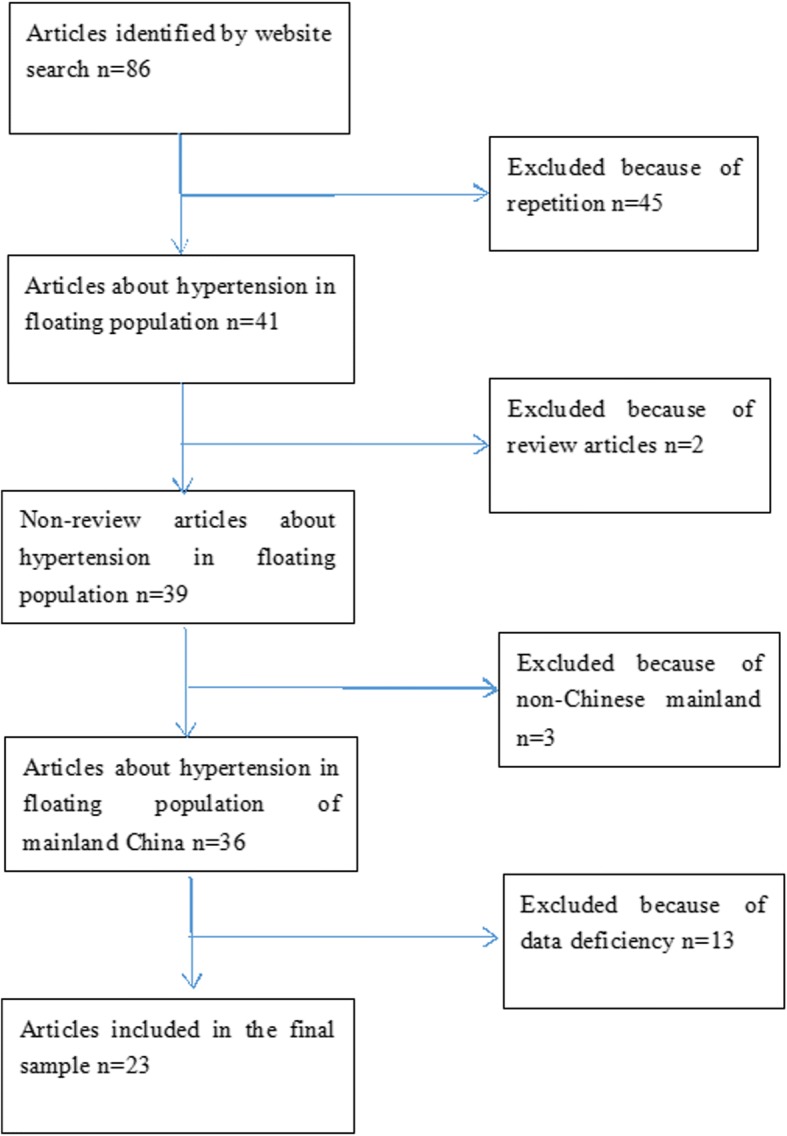
Quorum flow chart

The numbers of initial retrieval in the CNKI, Wan Fang data, PubMed, and the Web of Science were 12, 29, 10 and 16 respectively, and the final sample included 23 studies (Fig. 1). Subjects of the studies were distributed in different regions in China, different level units such as the provinces, municipalities and districts were included.

### Literature quality evaluation

As can been seen from Table 1, all the studies were conducted between 2010 and 2016, and the locations of subjects were distributed in different provinces, autonomous regions and municipalities in Mainland China. Among those selected studies, 1 study was conducted in the whole country, 8 studies in the administrative regions of province, 4 studies in the administrative regions of municipality and 10 studies in the administrative of district. The sample size ranged from 162 to 48,704. The scores of the included studies judged by the cross-sectional study quality evaluation criteria recommended by AHRQ show that they all have relatively good quality.

**Table 1:** Quality evaluation of included literature

First author	Published year	Sample size	Study area	Research type	Quality score
Xin Meng [24]	2015	1493	Jilin province	provincial	6
Tao Li [14]	2013	162	Akesu city,Xinjiang province	municipal	6
Xiaofei Wu [13]	2013	300	Kashi city, Xinjiang province	municipal	4
Qian Zhou [6]	2015	1500	Inner Mongolia province	provincial	8
Chunxia Liu [7]	2015	303	Haigang district, Qinghuangdao city	district	6
Chunxia Liu [8]	2015	300	Haigang district, Qinghuangdao city	district	6
Hua Li [9]	2015	2378	Hebei province	provincial	5
Lei Qiao [16]	2010	440	a district, Beijing	district	8
Xiuyun Sun [15]	2011	801	Chongwen district, Beijing city	district	4
Xiaoqin Feng [31]	2015	1990	Lvliang city,Shanxi province	municipal	5
Lixia Ma [19]	2014	610	Ningxia province	provincial	6
Yine Zhang [12]	2016	610	Xingqing district and Zhongning county,Ningxia province	district	6
Yaru Qin [11]	2015	303	Zhongning county, Ningxia province	district	5
Can Liu [10]	2016	304	Baohe district, Hefei city	district	6
Ying Deng [22]	2015	2373	Sichuan province	provincial	5
Yajun Meng [32]	2014	301	Huangshigang district, Huangshi city	district	4
Chuanhua Yu [17]	2016	1800	Hubei province	provincial	5
Tianjing He [18]	2016	1724	Hubei province	provincial	5
Donghui Jin [21]	2015	2088	Hunan province	provincial	6
Xiaohong Zhou [45]	2015	303	Xiacheng district, Hangzhou city	district	6
Kaixu Xie [46]	2014	1800	Tongxiang city, Zhejiang province	municipal	7
Yan Xu [20]	2015	1495	Jiangxi province	provincial	6
Ling Chen [47]	2015	302	Haicheng district, Beihai city	district	6

### The prevalence of hypertension in the floating population and comparison with general population

**Fig. 2:**
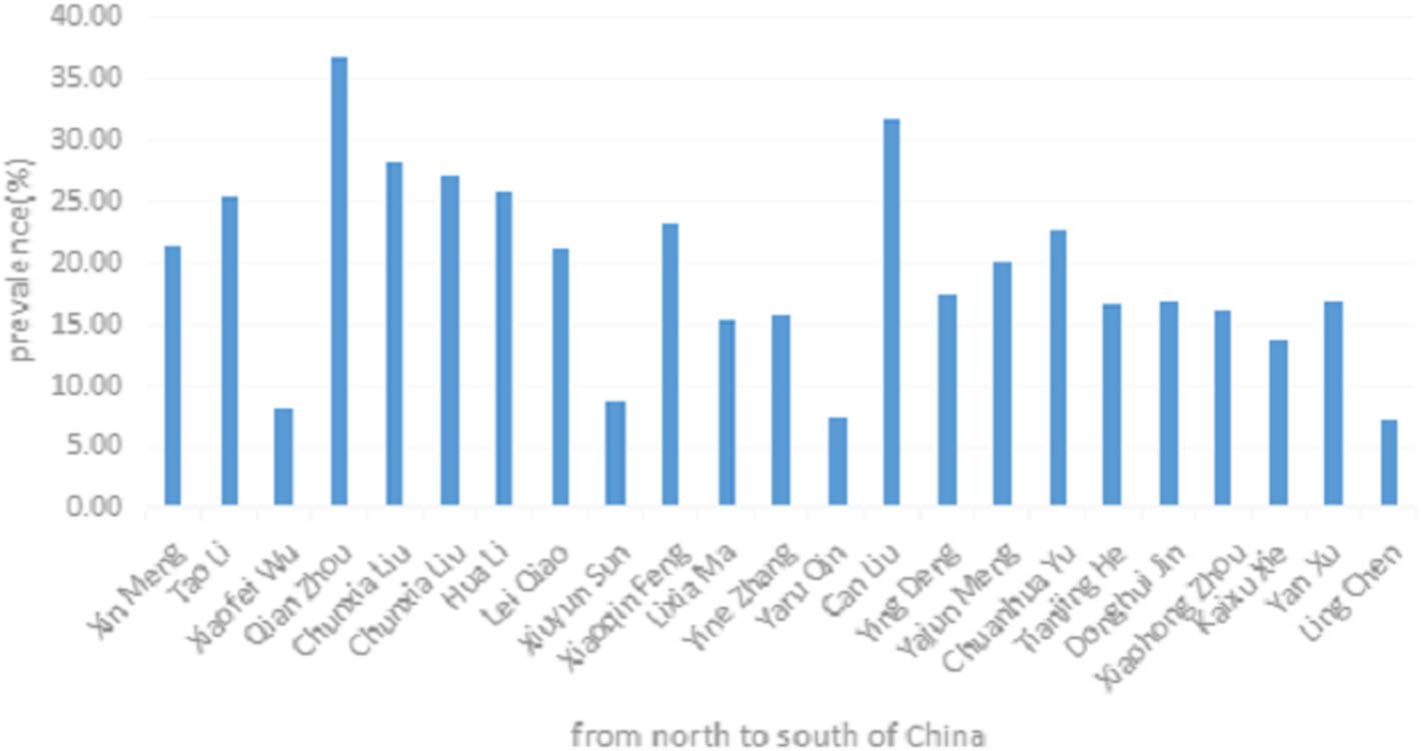
Prevalence of hypertension in floating population

According to a national survey conducted in China, 33.7% of Chinese adults were hypertensive patients [5].When it comes to the floating population, the prevalence of hypertension varies in different areas in China (Table 2). By comparison, we could find that except for the study in Inner Mongolia Province (36.5%) [6], most results show that the prevalence of hypertension in floating population is lower than that in national population in China. However, no study has done further analysis of the reasons for the difference. And the regions with higher prevalence of hypertension within the floating population are Hebei Province (27.00, 28.05 and 25.78%) [7–9] and Baohe district of Anhui Province (31.6%) [10], which are mainly located in the north of Mainland China. Through the analysis of those included studies, we also could find it shows a slight decreasing tendency in the prevalence of hypertension from north to south in China (Fig. 2).

**Table 2:** Summary of the prevalence of hypertension in included studies

First author	Study area	Prevalence
Xin Meng [24]	Jinlin province	21.37%
Tao Li [14]	Akesu city, Xinjiang province	25.31%
Xiaofei Wu [13]	Kashi city, Xinjiang province	8.00%
Qian Zhou [6]	Inner Mongolia province	36.50%
Chunxia Liu [7]	Haigang district, Qinhuangdao city	28.05%
Chunxia Liu [8]	Haigang district, Qinhuangdao city	27.00%
Hua Li [9]	Hebei province	25.78%
Lei Qiao [16]	a district, Beijing city	21.20%
Xiuyun Sun [15]	Chongwen district, Beijing city	8.70%
Xiaoqin Feng [31]	Lvliang city,Shanxi province	23.02%
Lixia Ma [19]	Ningxia province	15.30%
Yine Zhang [12]	Ningxia province	15.57%
Yaru Qin [11]	Zhongning county, Ningxia province	7.30%
Can Liu [10]	Baohe district,Hefei city	31.60%
Ying Deng [22]	Sichuan province	17.40%
Yajun Meng [32]	Huangshigang district, Huangshi city	19.93%
Chuanhua Yu [17]	Hubei province	22.70%
Tianjing He [18]	Hubei province	16.50%
Donghui Jin [21]	Hunan province	16.70%
Xiaohong Zhou [45]	Xiacheng district, Hangzhou city	16.17%
Kaixu Xie [46]	Tongxiang city, Zhejiang province	13.67%
Yan Xu [20]	Jiangxi province	16.80%
Ling Chen [47]	Haicheng district,Beihai city	7.28%

In addition, while the prevalence of hypertension in most studies are in the similar level, there also exist some inconsistent results in the same province. For example, the prevalence of hypertension in Zhongning county of Ningxia Province is only 7.3% [11], which is quite different from the research conducted by Yine Zhang in Ningxia Province (15.57%) [12]; and the prevalence in Kashi City of Xinjiang Province is 8.00% [13], lower than the prevalence of Akesu City of Xinjiang Province (25.31%) [14]^;^in Xiuyun Sun’s study [15], the prevalence in Chongwen District in Beijing City is 8.70%, which is also lower than the result of Lei Qiao’s result in Beijing (21.20%) [16]. Moreover, the prevalence in Chuanhua Yu’s study is 22.7% in Hubei Province [17], while it is only 16.5% in Tianjing He’s research [18]. This phenomenon might be attributed by the problem of sample selection.

### Comparison with local population

To find out the influencing factors of hypertension in floating population, a comparison of the prevalence between floating population and local residents is of great importance. Most studies show that the prevalence of hypertension in floating population is lower. In Hebei Province, the prevalence of hypertension in employed floating population is lower than that in local residents (27.86%) [8, 9]. Similar results can also be found in Hubei Province [17, 18], Ningxia Province [12, 19], Jiangxi Province [20] and Akesu City [14]. However, Qian Zhou’s study obtains an opposite result that the prevalence of hypertension in floating population in Inner Mongolia is higher than that in local residents above 15 years old in Hohhot City [6]. The inconsistence in results might be explained by the difference in demographic characteristics.

Researchers find that the deficient utilization of primary health service in floating population is also a notable problem. Xiuyun Sun’s study shows that the percentage of floating population who utilise the community health service is only 33.40% in Beijing [15], and the rate of community management of hypertension in floating population is 2.30% in Hunan Province [21], 13.30% in Ningxia Province [12], 25.00% in Hefei City [10] and 26.40% in Sichuan Province [22]. It is worth noting that 6.90% of the hypertensive floating patients have not realized the fact that they could use the community health management service without any charge [22].

### The gender difference in hypertension among floating population

According to the national survey about hypertension in China, the prevalence of hypertension in males (31.2, 95% CI 30.1–32.4%) is higher than that in females (28.0, 95% CI 27.0–29.0%) [23] . Similar results can also be found in most of the included studies about floating population [6, 10, 12, 16, 17, 19, 20, 22, 24]. In addition, Hua Li’s study also shows that the prevalence of hypertension in males is higher than that in females in floating population under 50 years old, but it reverse in floating population over 50 years old [9]. From a physiological perspective, endogenous sex hormones may play a certain role, and high level of oestrogen can help reduce the risk of hypertension in females [25]. Because the floating group is relatively young, and the average age of them is less than 40 years old [4], so the prevalence of hypertension in male floating population is higher than that in females. However, Yaru Qin’s study shows that the difference between prevalence of hypertension among men and women is not statistically significant [11].

### The age difference in hypertension among floating population

Increased age is an important risk factor of hypertension. According to a national survey, the age-specific prevalence of hypertension is 13.0% in young people (aged 20 to 44 years old), 36.7% in middle-aged people (aged 45 to 64 years old), and 56.4% in elderly people (aged≥65 years old) [26]. Although the floating population group is mainly composed of young people, the prevalence increases with ages, too. In Donghui Jin’s research [21], the prevalence of hypertension in floating population aged under 35 years old is 5.21%, and it increases to 28.39% in older group aged≥35 years old. Similarly, Xin Meng’s research shows the prevalence of hypertension in floating population group aged under 40 years old (14.2%) is significantly lower than that in older group aged≥40 years old (40.8%) [24]. The same conclusion can also be found in other related literature [6, 8–12, 16, 19, 20].

### The risk factor of hypertension among floating population

According to the global report on hypertension, unhealthy diet, harmful use of alcohol, lack of physical activities, excess weight and exposure to persistent stress are strongly related to hypertension [27]. Through the summary about relevant content in those selected literature, we choose the alcohol consumption and obesity as the major indicators of risk factors in floating population (Tables 3 and 4).

**Table 3:** Drinking rate of floating population in some included studies

First author	Study area	Drinking rate
Tao Li [14]	Akesu city, Xinjiang province	67.90%
Chunxia Liu [6]	Haigang district, Qinhuangdao city	66.33%
Xiaoqin Feng [31]	Lvliang city,Shanxi province	31.40%
Yajun Meng [32]	Huangshigang district, Huangshi city	46.51%

**Table 4:** Overweight and obesity rates of floating population in some included studies

First author	Study area	Overweight rate	Besity rate
Hua Li [9]	Hebei province	39.19%	16.99%
Xiuyun Sun [15]	Chongwen district, Beijing city	26.10%	5.90%
Xiaofei Wu [13]	Kashi city, Xinjiang province	24.00%	14.30%
Ling Chen [47]	Haicheng district,Beihai city	23.84%	7.80%

Guansheng Ma’s survey about the condition of alcohol consumption in general population shows that the rate of drinking is 21.0% [28]. All the included studies show that floating population tend to consume more alcohol. And the drinking rate in Akesu City of Xinjiang Province even reaches 67.90% [14], which greatly exceeds the national level. In the comparison with local residents, floating group is also likely to have more alcohol consumption. The rate of drinking in floating population in Qinhuangdao City of Hebei Province is 66.33%, which is higher than that in local residents (41.40%) [29]. The same result can also be seen in Shanxi Province, where the general drinking rate in local residents is 30.10% [30], a little lower than that in floating population in Lvliang City of Shanxi Province (31.40%) [31]. Moreover, the rate of drinking in floating population in Huangshi City (46.51%) [32] is even much higher than that in middle-aged and elderly local people (30.8%) [33], as well as male rural residents in Hubei Province (33.38%) [34]. Their high-intensive work or bad dietary habit might be a potential reason for the high drinking rate.

According to the national survey conducted in China, the rate of overweight is 29.3%(95% CI, 28.5–30.1%), and the rate of obesity is 10.6%(95% CI, 10.1–11.2%) [23]. In floating population, the overweight rate is 26.8%(95% CI,26.4–27.3%), and the obesity rate is 4.7%(95% CI,4.5–5.0%) [4], both are lower than the level of general population. While Hua Li’s study shows that the rates of overweight and obesity in floating population are 39.19 and 16.99% in Hebei Province [9], the results of most selected regional studies are close to the national level in floating population. Xiuyun Sun’s study reveals that the overweight rate and obesity rate in floating population in Beijing are 26.10 and 5.90% [15]. Xiaofei Wu’s study shows that the 24.00% of floating population are overweight and 14.3% of them are obese [13]. The rate of overweight in Beihai City of Guangxi Province is 23.84% [35], and the rate of obesity in Hubei Province is 7.80% [18] in floating population. The probable reason is that the floating population is mainly composed of relative young people who are engaged in labour-intensive work, which could help them effectively prevent overweight and obesity.

### The disease management of hypertensive floating population

Hypertension is an important public health problem all over the world because of its high prevalence and concomitant risk of cardiovascular and kidney disease [35, 36]. And it has also been identified as the leading risk factor of mortality and the third cause of disability-adjusted life-years [37]. Therefore, it is a great responsibility for the government to guarantee efficient long-term treatment for hypertensive patients and help them keep blood pressure well controlled. The rates of awareness, treatment and control are significant indicators to evaluate government’s function in the management system of chronic disease (Table 5).

**Table 5:** Management condition in some included studies

First author	Study area	Awareness rate	Treatment rate	Control rate
Lixia Ma [19]	Ningxia province	32.30%	\	40.00%
Qian Zhou [6]	Inner Mongolia province	44.20%	30.20%	\
Ying Deng [22]	Sichuan province	28.40%	\	\

According to Dongfeng Gu’s study, the awareness rate of hypertension in general Chinese adults is 44.7%, the treatment rate is only 28.2%, and the control rate is 81.0% [38]. The floating population are rarely involved in the local management system of chronic disease, so their condition is always much worse. Lixia Ma’s study shows that the awareness rate in floating population in Ningxia Province is 32.30%, control rate is 40.00% [19], both lower than that of the general population. In the comparison with local residents, Qian Zhou’s study in Inner Mongolia reveals that the rates of awareness, treatment are 44.20 and 30.20%, both lower than the level of local residents in Hulunbeier City (58.70 and 44.20%) [39]. Similar conclusion can also be seen in Sichuan Province, where the rate of awareness in floating population is 28.40% [22], lower than the level of local population within the province [40].

## Discussion

There exists a large gap between current disease management situation and the ideally acceptable state through above analysis. Major reasons of the worse management condition include the following three aspects. Firstly, most of the floating population have to migrate every year to make a living, which is quite inconvenient for the community health care center or other health department to carry out long-term and continuous monitoring and intervention towards them. And due to their tremendous liquidity, they are always neglected by the basic public health service allocation system, which is mainly based on the number of residents within the jurisdiction to make the financial budget. In that case, the local primary health care centers don’t have the motivation and capacity to provide service to floating population. Secondly, the number of floating population in China is still increasing rapidly, which would pose a great challenge to the hypertension prevention and management system. In 1990, the number of floating population in China was nearly 22 million, and then the size doubled in the five years from 1990 to 1995 [41]. By 2000, it had reached 121 million, representing 10% of China’s total population at that time [42]. However, the supply capacity of health service in community health service centers, township hospitals, and other basic health institutions can not meet the floating population’s requirements in a short time, so more practitioners and related facilities are in need. Thirdly, floating population are always known to be engaged in 3D jobs (dirty, difficulty, and dangerous), and they have to endure lower wages, and poor housing conditions [43, 44]. All these poor living factors could be harmful for their health. In order to finish the labor-intensive job, they have to spend more time and energy, and thus they don’t have enough free time to care about physical health or receive health education and blood pressure management. Moreover, because of the slender income, they are also unable to enjoy timely health care service and effective medication treatment.

## Conclusion

This review contains all the literature about hypertensive floating population in Mainland China. The major result is that the prevalence of hypertension in floating population is generally lower than that in general population and local residents in China, except for some regions. In addition, males and aging floating groups are more likely to suffer from hypertension. Compared with general population, the drinking rate in floating population is much higher, but the rates of overweight and obesity are roughly lower. Finally, the disease management of floating population cannot meet the requirements and still needs to be improved. So the major problem of floating population is focused on their unhealthy lifestyle (drinking) and deficient disease management.

The results indicate that the potential threat brought by hypertension in floating population is imponderable. To achieve the “primary health care for all” health strategic objectives, the relevant departments should undertake the responsibilities to care about the floating population. In order to satisfy the increasing health service need of floating population, some relevant effective measures and policies are urgent to be carried out.

Recommendations are as follows: Firstly, increasing the fund and facility support for public health service system so as to improve their service delivery ability and reduce medical burden of floating population. Secondly, in order to strengthen their health awareness and improve compliance to disease management, it is also necessary to enforce the health education and unhealthy lifestyle intervention towards floating population by health lectures or follow-up, including limiting alcohol consumption, enhancing physical exercise and maintaining a healthy weight.

### Study limitations

The strength of this article is the complete inclusion of all published studies about the hypertensive patients in floating population of Mainland China, and it is the only review on this topic in the literature. The limitation of this study is the relatively small sample size in some researches.
